# Comparisons make faces more attractive: An ERP study

**DOI:** 10.1002/brb3.2561

**Published:** 2022-05-12

**Authors:** Shangfeng Han, Jie Hu, Jie Gao, Jiayu Fan, Xinyun Xu, Pengfei Xu, Yuejia Luo

**Affiliations:** ^1^ Shenzhen Key Laboratory of Affective and Social Neuroscience Center for Brain Disorders and Cognitive Sciences School of Psychology Shenzhen University Shenzhen China; ^2^ School of Psychology Sichuan Center of Applied Psychology Chengdu Medical College Chengdu China; ^3^ Beijing Key Laboratory of Applied Experimental Psychology National Demonstration Center for Experimental Psychology Education (BNU) Faculty of Psychology Beijing Normal University Beijing China; ^4^ Center for Emotion and Brain Shenzhen Institute of Neuroscience Shenzhen 518057 China; ^5^ College of Teacher Education Qilu Normal University Jining China; ^6^ The Center of Brain Science and Visual Cognition Kunming University of Science and Technology Kunming China

**Keywords:** EEG, emotion, facial attractiveness, microstate, narcissism

## Abstract

Facial attractiveness judgment largely depends on the characteristics of the facial structure and the personality of the observer. However, little is known about the influence of contextual variations on facial attractiveness. In this electroencephalogram study, participants judged the attractiveness of faces presented individually or in pairs with either a higher‐attractive face (HAF) or lower‐attractive face (LAF). The attractiveness judgment rating of the target face was significantly higher when presented in pairs with HAFs or LAFs than when presented individually and was accompanied by a larger late positive complex. These results suggest that contextual faces enhance the attractiveness judgment of target faces. Microstate analyses revealed that the global field power (GFP) of state 3 was significantly correlated with the attractiveness judgment in the HAF condition whereas the GFP of state 2 was significantly correlated with the attractiveness judgment in the LAF condition. Interestingly, the GFP of state 2 mediated the relationship between narcissism and facial attractiveness judgment in the context of LAFs. Source location analyses showed that states 3 and 2 activated the superior and middle frontal gyrus, which are involved in emotion processing. Our findings suggest that facial attractiveness can be enhanced by contextual comparison with other faces, subject to personality of the observer.

## Graphical abstract

By using the electroencephalogram (EEG), the spatiotemporal dynamics of the brain were measured when faces were paired with high and low attractiveness faces. Our results show that the face was judged more attractive than it presents alone when it appears with others faces. Our findings contribute to the understanding of neurocognitive mechanisms and processes of facial attractiveness.

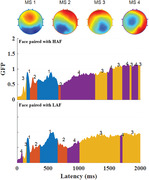

## 1 INTRODUCTION

Facial attractiveness is an important social attribute (Todorov et al., 2015) and has been considered a health cue associated with good genes (Scheib et al., 1999). In individuals with high facial attractiveness, survival is likely based on the evolutionary perspective (Little et al., 2011). Facial structure, including averageness, symmetry, sexual dimorphism, and texture, is the main factor that influences facial attractiveness judgment (Little, 2014). Although facial attractiveness is shown to be stable (Rhodes et al., 2001), accumulating evidence suggests that facial attractiveness judgment can change as the context varies (Han et al., 2020; Kedia et al., 2014). Faces often appear in groups in daily life; however, it remains unclear how facial attractiveness judgment changes in comparison with other faces.

Facial attractiveness varies according to context (Forsythe et al., 2014). Previous studies showed that people are more attractive in a group than in isolation (Walker & Vul, 2014), and the spatial arrangement of the faces in the group does not influence this phenomenon (Carragher et al., 2018). While an assimilation effect of consistency between the attractiveness ratings of a target face and contextual faces has been previously observed (Geiselman et al., 1984), a contrast effect of opposite attractiveness ratings between a target face and contextual faces has also been reported (Lei et al., 2020). The inconsistency between assimilation and contrast might be explained by the perceiver's concerns. A perceiver is focused on similarities or differences between a target and its context, leading to assimilative or contrastive judgments (Mussweiler, 2001). Therefore, it is important to control the differences between contextual and target faces.

Individual differences have been shown in preferences for facial attractiveness (Zhang et al., 2014), especially for faces with moderate attractiveness (Han et al., 2018). For instance, individuals with high narcissism, a personalized characteristic associated with an excessively positive self‐concept but low empathy or parental density (Campbell et al., 2011), tend to overestimate their attractiveness (Holtzman & Strube, 2010). The more attractive an individual perceives themselves to be, the less processing resources they appear to devote to the unattractive faces in their environment (Morgan & Kisley, 2014). Consequently, personality differences, such as narcissism, may be associated with attractiveness judgment.

In this study, we aimed to examine the influences of contextual faces on facial attractiveness judgment and its neural mechanisms, as well as the potential role of narcissism. A target face alone or paired with a higher (HAF) or lower attractive face (LAF) was presented. Given that facial attractiveness is more important for men than for women (Buss & Schmitt, 2019) and female facial attractiveness has a greater reward value for men (Cloutier et al., 2008), we recruited only male participants to judge the attractiveness level of a target female face. To examine the underlying neural processes, we measured event‐related brain potentials, focusing on the late positive complex (LPC), which is associated with the evaluation of facial attractiveness (Schacht et al., 2008). We conducted microstate analyses to identify spatiotemporally dynamic changes during the processing of facial attractiveness and the corresponding functional states of the brain (Michel & Koenig, 2018). In an assimilation effect, the attractiveness of the target face would be higher or lower when paired with an HAF or LAF, than when it appeared alone. In contrast, a lower rating of the target face when paired with an HAF or a higher rating of the target face when paired with an LAF than that when it appeared alone would support the hypothesis of the contrast effect.

## 2 MATERIALS AND METHODS

### 2.1 Participants

Twenty‐three men, aged between 22 and 29 years (*M *= 24.43, *SD *= 1.88), participated in the study. All participants reported that they were heterosexual and had no history of neurological illness. They received 40 Yuan after completing the task. Informed consent was obtained from all participants, and the study was approved by the local institutional ethics committee.

### 2.2 Material

We selected 300 female faces from the Internet. Photoshop CS6 was used to adjust all images to the same size (141 × 197 pixels) and position the black‐and‐white oval faces, and SHINE toolbox (Willenbockel et al., 2010) was used to match the brightness (100 cd/m^2^). Thirty‐two students who did not participate in the formal experiment judged the attractiveness of the processed faces on a 7‐point scale, 1 = “very unattractive” to 7 = “very attractive.” According to the score, 40 HAFs and LAFs and 80 middle‐attractive faces (MAFs) were selected. The MAFs were randomly divided into Group 1 and Group 2 and matched with HAFs and LAFs, according to the score order, respectively. There were 40 pairs of HAFs and MAFs and 40 pairs of LAFs and MAFs. The spatial location of the target face appeared randomly on the left or right side of the screen when there was a paired face presented, but at the center of the screen when it was presented alone. One‐way repeated‐measures analysis of variance (ANOVA) was used to identify differences in facial attractiveness among groups. The results showed that there were significant differences between HAFs (*M *= 4.24, *SD *= 0.36), MAFs (Group 1 of MAFs: *M*_1_*__* = 2.87, *SD *= 0.31; Group 2 of MAFs: *M*_2 _= 2.84, *SD *= 0.29), and LAFs (*M *= 1.51, *SD *= 0.08) (*F*(3156) = 629.46, *p *< .001, η_p_^2^_ _= 0.92). The scores of HAFs were significantly higher than those of MAFs and LAFs (*p *< .001). The scores of the two MAF groups were significantly higher than those of the LAFs (*p *< .001). There were no significant differences between the two MAF groups (*p = *.62). Furthermore, there was no significant difference between the high minus middle attractiveness ratings and the middle minus low attractiveness ratings (*t*(39) = 1.24, *p *= .22).

### 2.3 Questionnaire

The Narcissistic Personality Inventory (NPI) consists of 40 items in a forced‐choice format (Raskin & Terry, 1988) and is used to measure narcissism (Hewitt & Flett, 1991; Maxwell et al., 2011). Participants rated themselves on each item using a 5‐point response scale ranging from 1 (*strongly disagree*) to 5 (*strongly agree*). Our data showed good internal consistency of the scale (Cronbach's alpha = .96). Higher scores indicated higher degrees of narcissism.

### 2.4 Procedure

The experimental procedure was performed using E‐Prime 3.0. The formal procedure consisted of two blocks. The first block was the paired face block; following a 500‐ms fixation “+,” the paired faces appeared randomly for 2000 ms. Participants only needed to pay attention to the two faces. Next, an arrow to the left or right was displayed at the center of the screen, which was used to indicate to the participants to judge the face in the next step (the arrow always points to the MAF). Next, the participants were asked to press keys “1” (very unattractive) to “7” (very attractive) to judge the attractiveness of the face pointed by the arrow within 5000 ms. Finally, an empty screen appeared for a random period of time from 800 ms to 1200 ms after the participants had made their judgments. After a short rest period, the participants attempted the second block that was the single face block. After the 500 ms fixation “+” disappeared, the MAFs appeared for 2000 ms. Subsequently, participants were asked to press the keys to judge the facial attractiveness in the same manner as before. Finally, an empty screen randomly appeared for 800 to 1200 ms. The order of the block was balanced according to the participants.

**FIGURE 1:**
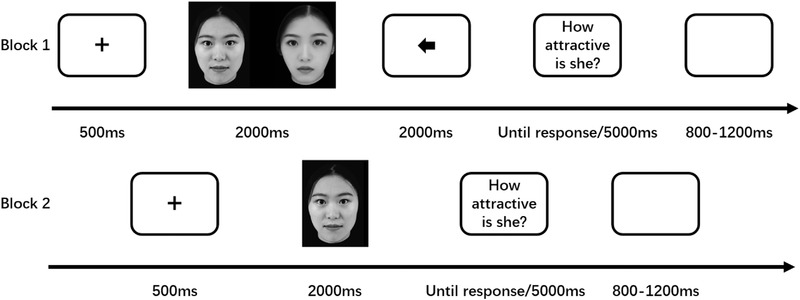
Schematic of the experiment procedure. In block 1, participants were asked to judge the face where the arrow pointed. In block 2, participants were asked to directly judge the face presented

Four exercise trials were conducted before the formal experiment to familiarize the participants with the experimental procedure. There were 160 trials in the paired face block (80 paired faces, MAFs appeared once each on the left or right randomly). There were 80 trials in the single face block, and the participants were asked to judge one of 80 MAFs randomly. To control the effect of the physical factors of the faces, participants judged the faces were the same in both the paired face block and the single face block. The specific procedure is shown in Figure 1.

### 2.5 Electroencephalography data acquisition and analysis

Electroencephalography (EEG) was recorded from 64 channels according to the 10‐20 International System by the Brain Products system of Germany. The online reference was the FCz. The EEG signal was recorded with a band‐pass of 0.01–100 Hz and digitized at 500 Hz. The impedance was maintained at 5 kΩ. The offline analysis used EEGLAB 19.0 (Delorme & Makeig, 2004). A common average reference was applied, and the EEG data were filtered from 0.01 Hz to 30 Hz. The arrow appeared at staged epochs that spanned from 0 to 2000 ms, and the judgment epochs spanned from 200 ms before to 1000 ms after the judgment display onset. Eye blinks were removed using an independent component analysis method. Finally, artifacts beyond ±80 μV were removed.

### 2.6 Data analysis

To reveal the influence of contextual variation on facial attractiveness, we used a one‐way ANOVA (Context: HAFs vs. LAFs vs. no face) to analyze behavioral and event‐related potential data. For the LPC component, the mean amplitude was calculated between 300 and 500 ms including electrodes CPz and Pz. When the data did not conform to the hypothesis of the spherical test, Greenhouse–Geisser epsilon correction was applied, and the Bonferroni correction was used for post hoc comparisons.

To identify the brain dynamic processing for the influence of contextual variation on facial attractiveness, microstate analysis was conducted at the stage when the arrow appeared using the Microstate EEGLAB toolbox version 1.0, a plug‐in for EEGLAB software (Poulsen et al., 2018). The epochs were between 0 and 2000 ms after arrow onset. After loading all the data, we selected the preprocessed data for the microstate segmentation. Consistent with previous studies (Michel & Koenig, 2018; Pedroni et al., 2017), we selected four states for further analysis. The next step was to fit the microstate prototypes back to the averaged EEG data of each participant. To reduce these spurious influences, the microstate labels were temporally smoothed after back‐fitting. Finally, the indices of the microstates, including global field power (GFP), occurrence, duration, and coverage, were calculated. Specifically, GFP reflects the strength of the average global activation during a given microstate. Occurrence is defined as the average number of times per second a microstate is dominant. The duration reflects the average duration of a microstate. Coverage is the fraction of time a given microstate is active (Poulsen et al., 2018).

To reveal the relationships among the brain temporal dynamic parameters of microstates, behavioral judgments, and narcissism, correlational analyses and mediation analyses were performed using PROCESS macro on SPSS 22.0 (IBM Corp., Somers, New York, USA; http://www.spss.com). Specifically, the narcissism score was the independent variable, the microstate parameters were the mediating variables, and the behavioral judgments were the dependent variables. Correlation analysis was conducted between the independent, mediating, and dependent variables. Only when the mediating variable significantly correlated with both the independent and dependent variables was the mediation analysis continued.

The brain structures underlying the microstates associated with facial attractiveness were identified by conducting a source location analysis. The intracerebral sources of the microstates were estimated using the sLORETA software (Pascual‐Marqui, 2002), which localizes both the superficial and deep brain structures (Pizzagalli et al., 2004). We used the MNI152 template as the head model, and the intracerebral volume was partitioned into 6239 voxels at 5‐mm spatial resolution.

## 3 RESULTS

### 3.1 Behavior results

Facial attractiveness judgment score analyses yielded the main effect of context (*F*(2, 44) = 12.00, *p* < .001, η^2^_p_ *= *0.35). Post hoc analysis revealed that the judgment rating of an MAF paired with an HAF (*M *= 3.49, *SE *= 0.16) was significantly higher than that of a single MAF (*M *= 3.28, *SE *= 0.18; *p *= .03). The judgment rating of an MAF paired with an LAF (*M *= 3.80, *SE *= 0.20) was significantly higher than that of a single MAF (*p* < .001) and that of an MAF paired with an HAF (*p *= .02). These results indicate that regardless of whether the facial context is an HAF or an LAF, the attractiveness judgment of the target face is improved.

### 3.2 Microstate results

**FIGURE 2:**
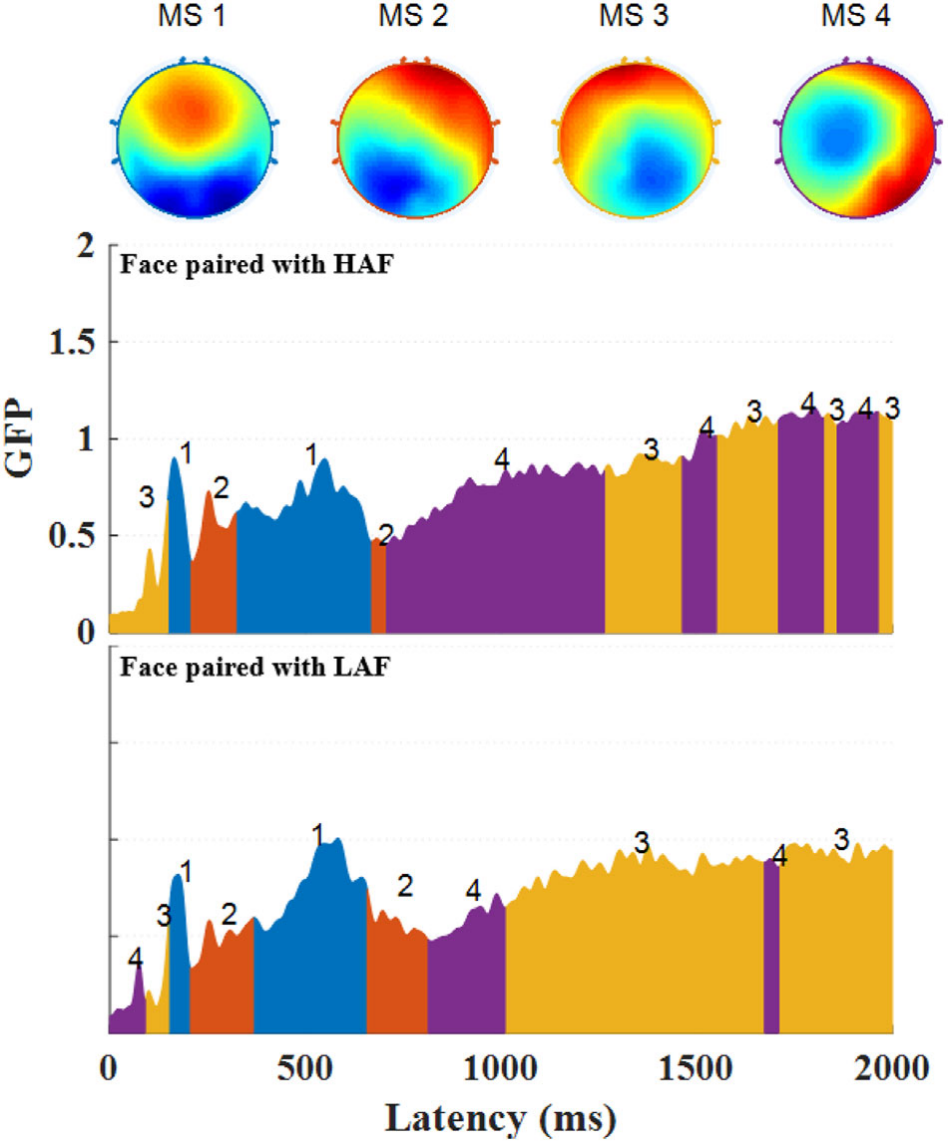
Four microstate (MS) prototypes (from 1 to 4) and their labels (top part of the photo). Segment maps of face that paired with high‐attractive face (HAF; middle part of the figure) and LA face (LAF; bottom part of the figure)

The mean values and standard deviations of microstate parameters, including average GFP, duration, occurrence, and coverage, are reported in Table 1. The mean prototype maps of the four microstate classes explained 58.86 ± 5.46% of the total variance (see Table 1 and Figure 2).

**TABLE 1:** Descriptive statistical table of microstate (MS)

	HA face paired face (*M* ± *SD*)				LA face paired face (*M* ± *SD*)			
MS	GFP (μV)	Coverage (%)	Duration (ms)	Occurrence (s)	GFP (μV)	Coverage (%)	Duration (ms)	Occurrence (s)
A	0.58 ± 0.16	32.33 ± 10.10	77.49 ± 22.01	4.33 ± 1.13	0.57 ± 0.16	35.27 ± 10.52	76.56 ± 17.53	4.67 ± 1.30
B	0.51 ± 0.12	23.60 ± 11.00	60.30 ± 15.87	3.78 ± 1.01	0.48 ± 0.14	24.99 ± 9.69	62.43 ± 16.97	4.00 ± 1.24
C	0.54 ± 0.15	23.13 ± 11.76	66.97 ± 19.07	3.89 ± 1.42	0.51 ± 0.17	22.79 ± 10.18	62.70 ± 14.53	3.57 ± 1.11
D	0.43 ± 0.15	17.94 ± 8.94	55.95 ± 9.88	3.15 ± 1.21	0.43 ± 0.15	16.96 ± 8.80	54.20 ± 10.83	3.11 ± 1.55

### 3.3 Correlation and mediation analysis

In the paired HAF condition, the narcissistic scores (range from 47 to 188, *M *= 112.74, *SE *= 36.42) were significantly negatively related to the average GFP of state 2 (*r = *−0.46, *p *= .03), and the average GFP of state 3 was significantly positively related to the attractiveness judgment in the HAF paired condition (*r = *0.43, *p *= .04). In the LAF paired condition, the narcissistic scores were significantly negatively related to the average GFP of state 1 (*r = *−0.47, *p *= .02) and state 2 (*r = *−0.45, *p *= .03); the average GFP of state 2 was significantly positively related to the attractiveness judgment in the LAF paired condition (*r = *0.42, *p *= .05, see Table 2 and Table 3).

**TABLE 2:** Descriptive statistical table of correlation analysis results in HAF condition

		1	2	3	4	5	6
1 Narcissism	*p*						
	*r*	—					
	*df*						
2 GFP 1	*p*	−.39					
	*r*	.06	—				
	*df*	22					
3 GFP 2	*p*	−.46*	.54**				
	*r*	.03	.01	—			
	*df*	22	22				
4 GFP 3	*p*	−.01	.52	.29			
	*r*	.96	.01	.18	—		
	*df*	22	22	22			
5 GFP 4	*p*	−.13	.69**	.41	.80**		
	*r*	.57	.00	.06	.00	—	
	*df*	22	22	22	22		
6 FAJ	*p*	.06	.25	.20	.43*	.35	
	*r*	.78	.26	.36	.04	.11	—
	*df*	22	22	22	22	22	

Abbreviations: FAJ, facial attractiveness judgment; GFP, global field power.

* *p < *.05, ** *p < *.01,

**TABLE 3:** Descriptive statistical table of correlation analysis results in LAF condition

		1	2	3	4	5	6
1 Narcissism	*p*						
	*r*	—					
	*df*						
2 GFP 1	*p*	−.47*					
	*r*	.02	—				
	*df*	22					
3 GFP 2	*p*	−.45*	.52*				
	*r*	.03	.01	—			
	*df*	22	22				
4 GFP 3	*p*	−.13	.54**	.39			
	*r*	.56	.01	.08	—		
	*df*	22	22	22			
5 GFP 4	*p*	−.34	.76**	.51*	.45*		
	*r*	.12	.00	.01	.03	—	
	*df*	22	22	22	22		
6 FAJ	*p*	.02	.20	.42*	.33	.17	
	*r*	.92	.35	.05	.13	.44	—
	*df*	22	22	22	22	22	

Abbreviations: FAJ, facial attractiveness judgment; GFP, global field power.

* *p < *.05, ** *p < *.01.

**FIGURE 3:**
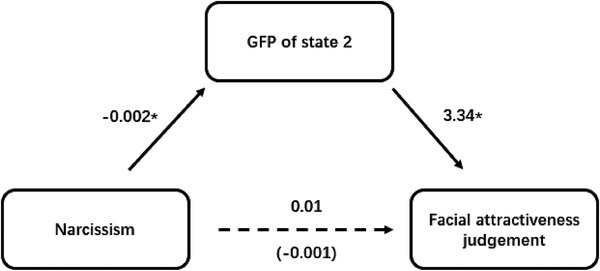
The mediation model. Modulation of narcissism on facial attractiveness was mediated by GFP of state 2 when target face was compared with lower attractive one. *p < .05

To explore whether the relationship between narcissism and facial attractiveness was mediated by state 2 in the LAF condition, we conducted the Preacher and Hayes’ bootstrapping analysis using PROCESS macro. With 5000 bootstrap samples, the 95% confidence interval (CI) of the mediating effect was estimated for the mediating effect test. We used the narcissism score as the independent variable, facial attractiveness score on pairing with LAFs as the dependent variable, and the GFP of state 2 in the LAF paired condition as the mediating variable to test the mediating effect. The results showed that narcissism can significantly predict the GFP of state 2 (*β *= −0.06, *SE = *0.03, *t*(21) = −2.31, *p *= .03). The GFP of state 2 also significantly predicted the facial attractiveness judgment (*β *= 3.34, *SE = *1.46, *t*(21) = 2.29, *p *= .03). Under 95% confidence interval (CI), the indirect effect of narcissism on facial attractiveness judgment did not include zero (LLCT = −0.83, ULCT = −0.02). A value of zero was included in the 95% CI of the direct effect (LLCT = −0.24, ULCT = 0.63). These results indicated a complete mediating effect of state 2 on the association between narcissism and facial attractiveness judgment in the LAF condition (see Figure 3).

### 3.4 Source localization analysis

**FIGURE 4:**
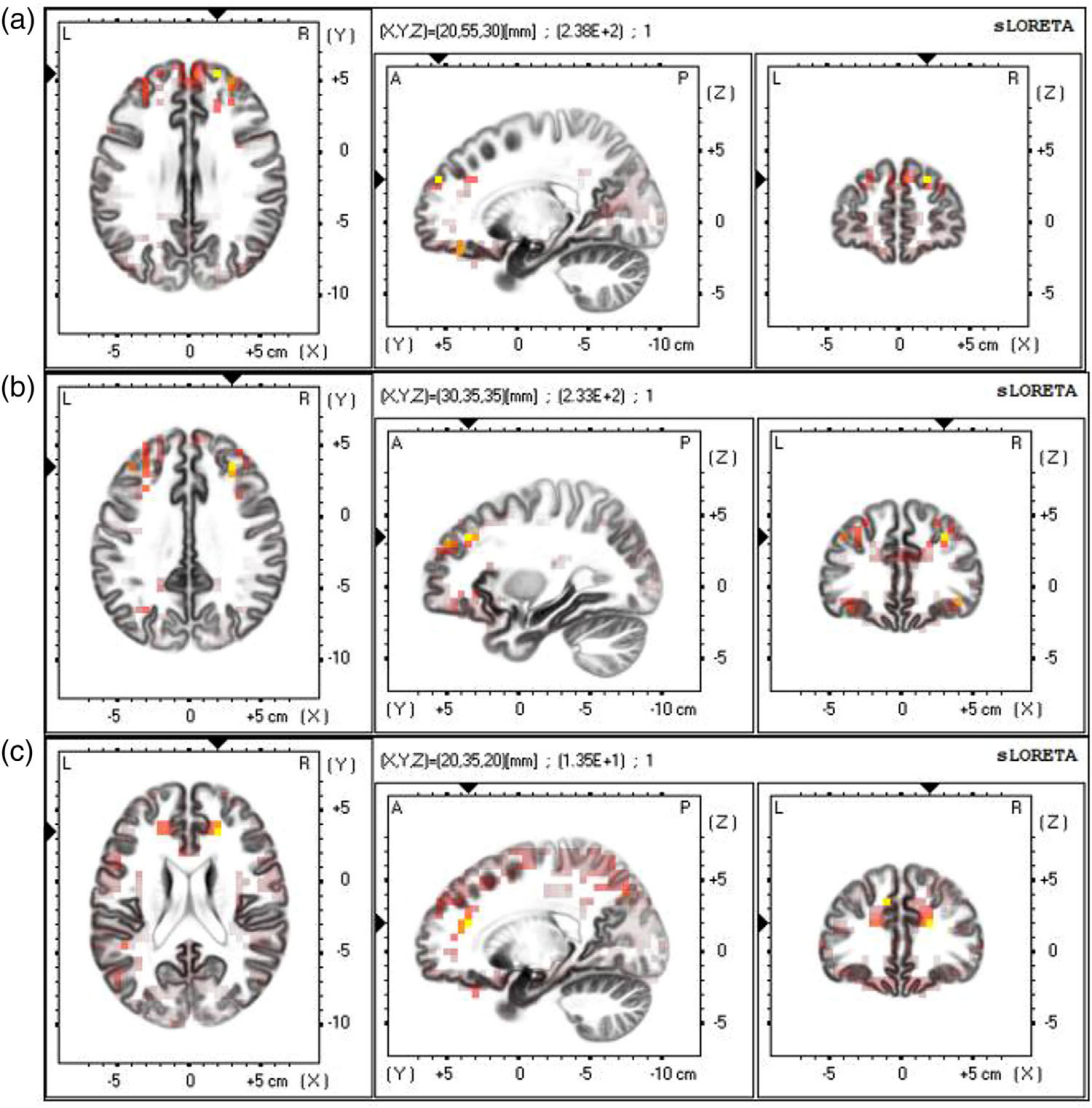
(A) Superior frontal gyrus and (B) middle frontal gyrus were activated for the microstate 3 in the HA faces paired condition. (C) Middle frontal gyrus were activated for the microstate 2 in the LA faces paired condition

The whole‐brain voxel‐by‐voxel one‐simple *t* test (corrected for multiple testing; Nichols & Holmes, 2002) showed that the peak voxel of state 3 in the HAF condition was located in the Brodmann area 9: the superior frontal gyrus (MNI coordinates: X = 20, Y = 55, Z = 30) and middle frontal gyrus (MNI coordinates: X = 30, Y = 35, Z = 35), *t*(22) > 5.78, *p* < .01. The peak voxel of state 2 in the LAF condition was also located in the Brodmann area 9: the middle frontal gyrus (MNI coordinates: X = 20, Y = 35, Z = 20; *t*(22) > 5.64, *p* < .01; see Figure 4).

### 3.5 ERP results

**FIGURE 5:**
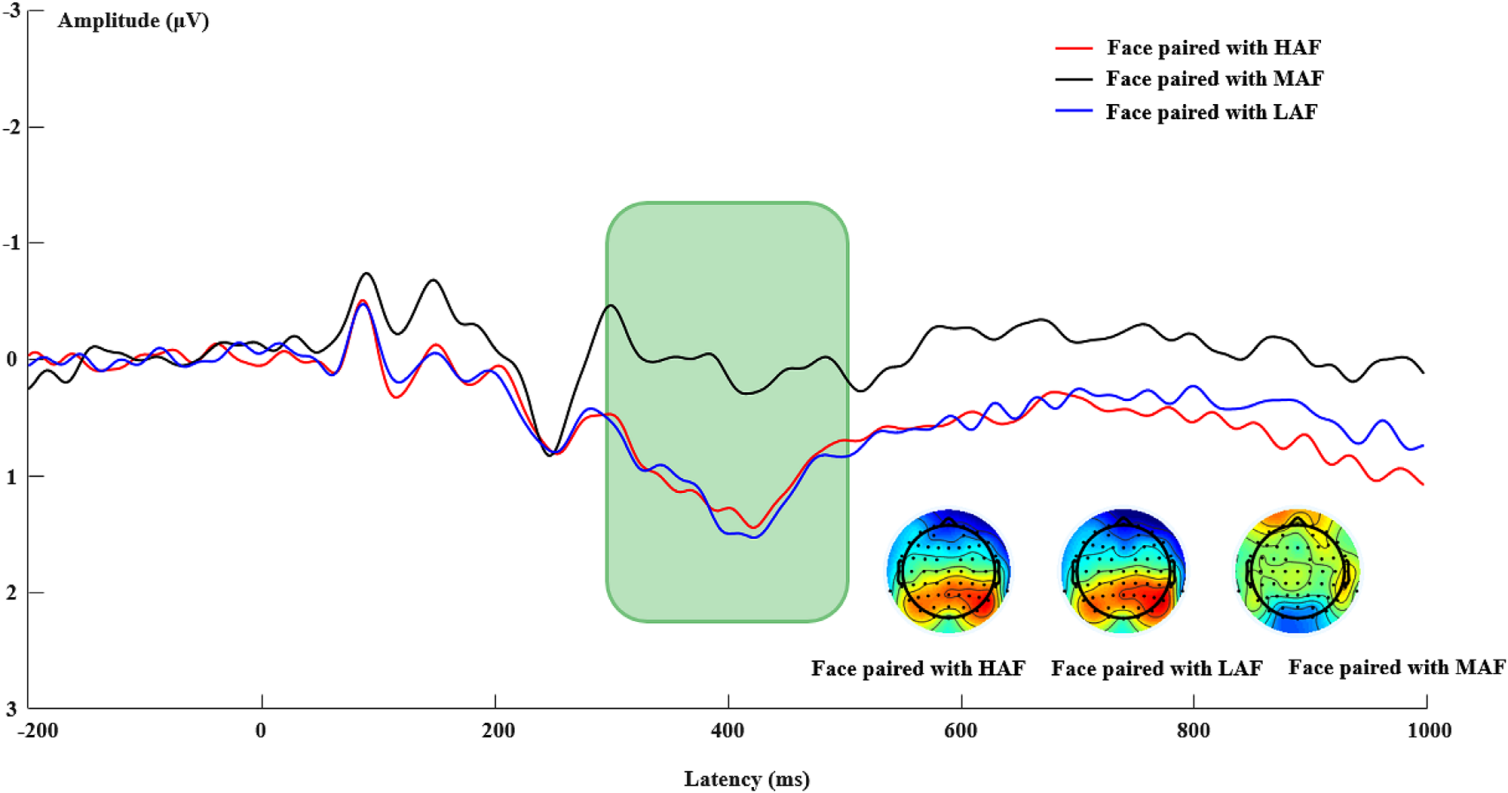
Grand‐averaged event‐related potential waveforms are shown for LPC. CPz and Pz were selected for LPC (shaded 300–500‐ms time window) waveforms. HAF, high‐attractive face; LAF, low‐attractive face; MAF, middle‐attractive face

The mean amplitude of the LPC analyses revealed a main effect of attractiveness (*F*(2, 44) = 14.01, *p* < .01, η_p_^2^ *= *0.39). Post hoc analysis showed that both the HAF (*M *= 1.05, *SE *= 0.23) and LAF (*M *= 1.10, *SE *= 0.23) pairs evoked a larger LPC than the unpaired face (*M *= 0.04, *SE *= 0.30), *p*s < 0.01. There was no significant difference in the evoked LPC between the HAF and LAF pairs, *p *= .63 (see Figure 5).

## 4 DISCUSSION

This study examined the influence of contextual faces on facial attractiveness judgments and potential individual differences. The results show an assimilation effect when faces are paired with HAFs, while a contrast effect occurs when faces are paired with LAFs. Importantly, the prefrontal areas associated with emotional processing are involved in facial attractiveness judgment.

Although there are numerous studies on the mechanism (e.g., familiarity, traits, ensemble perception) of facial attractiveness judgment (Carr et al., 2017; Han et al., 2018; Luo & Zhou, 2018), none have addressed the role of emotion. Emotion processing in facial perception has been widely studied. Attractive female faces are recognized as happier faces than unattractive faces (Lindeberg et al., 2019). Attractive faces activate the reward brain system, eliciting positive emotions (Chatterjee et al., 2009; North et al., 2010; Winston et al., 2007). Therefore, emotion processing may be pivotal in judging facial attractiveness.

A face was more attractive when presented paired with HAFs, supporting the hypothesis of the assimilation effect. One explanation for this might be emotion‐driven attention‐biased attractiveness judgment. A series of studies has shown that emotional stimuli are more likely to capture attention (Öhman et al., 2001; Schupp et al., 2006). A recent study found that in a crowd of attractive faces, participants paid more attention to happy faces (Mertens et al., 2020). Attractive faces capture attention more easily (Sui & Liu, 2009), especially considering the observations of male perceivers on female faces (Duncan et al., 2007). Increased attention, in turn, enhances facial attractiveness (Störmer & Alvarez, 2016). In this study, the HAFs may have evoked positive emotions that could be generalized to the target face and, in turn, improved the attractiveness of the target face.

Strategies and perceptual mechanisms of attractiveness judgments between HAFs and LAFs might differ (Thiruchselvam et al., 2016). For instance, the judgment of an attractive face depends on the contrast of the eyes, while the same is not true for an unattractive face (Störmer & Alvarez, 2016). In contrast to the assimilation effect evoked by an HAF, there was a contrast effect when the face was paired with an LAF. LAFs are associated with negative emotions, which perceivers wanted to avoid (Mertens et al., 2020). When paired with LAFs, the target face might be perceived as more positive. As a result, the face was judged to be more attractive than when it appeared alone. Interestingly, MAFs paired with LAFs were even more attractive than when paired with HAFs. One possible reason is that when people judge the target stimulus, they always compare it with the context, which serves as a reference (Furl, 2016). Although an assimilation effect occurred when the target face, i.e., an MAF was paired with an HAF, the reference was the HAF, which might have caused the judgment rating to not be too high. However, when the target face, i.e., an MAF was paired with an LAF, the reference was the LAF, and participants tended to assign a higher score. Another explanation may be the positive‐negative asymmetry, which is the greater impact of negative stimuli than of positive stimuli on people (Peeters & Czapinski, 1990). The LAFs evoked negative emotion (Mertens et al., 2020) and the HAFs evoked positive emotion (Chatterjee et al., 2009). Compared with the positive emotion, the negative emotion evoked may have improved the judgment of the target face, which may be one reason that the contrast effect was stronger when paired LAFs than the assimilation effect when paired HAFs.

Activation of prefrontal areas that are related to emotion regulation suggests that affective processing is involved in the modulation of contextual comparison of facial attractiveness. While greater activation of the superior frontal gyrus has been shown in crowd emotions than in individual emotion conditions (Im et al., 2017), activation of the middle frontal gyrus has been observed in processing attractiveness of repeated faces (Han, Liu, Gan et al., 2020). A previous study showed that activation of the middle frontal gyrus was associated with negative emotion regulation (Navas et al., 2017). Therefore, we speculated that participants may extract the emotion from contextual faces, which can affect the attractiveness judgment of the target faces.

Microstates reflect rapid switching between series of cognitive processing activities among different brain areas (Khanna et al., 2015; Michel & Koenig, 2018). The GFP represents the strength of the average global activation during spatiotemporal dynamic changes of microstates (Poulsen et al., 2018). The GFP of a specific state associated with the judgment of facial attractiveness in both the HAF and LAF conditions indicates the spatiotemporal dynamic changes during the processing of facial attractiveness. This idea is also supported by previous studies that suggest that the processing of facial attractiveness changes with time (Han, Liu, Gan et al., 2020) and context (Carragher et al., 2021). Interestingly, the GFP of state 2 mediated the relationship between narcissism and facial attractiveness judgment in the context of LAFs. To identify the brain source of the state related to the judgment of facial attractiveness, source location analysis was conducted. The results showed that emotion‐related brain areas (the superior frontal gyrus and middle frontal gyrus) were activated, suggesting that the dynamic emotional processing‐related state plays an important role in the influence of contextual variations on facial attractiveness.

As expected, narcissism was associated with judgments of facial attractiveness. Specifically, the modulation of narcissism on facial attractiveness was mediated by the whole‐brain activation intensity when the target face was compared with the LAF. While attractive judgment is modulated by both the attractiveness of a target face and the perceiver's attractiveness level (Morgan & Kisley, 2014), narcissists tend to exaggerate their attractiveness (Holtzman & Strube, 2010). The attractiveness judgment of narcissists is consistent with the matching hypothesis that people choose a partner with comparable attractiveness (Kalick & Hamilton, 1986). Narcissistic individuals might consider their levels of attractiveness to be high. Thus, they may recruit fewer brain resources in the attractiveness judgment of LAFs and provide lower ratings.

## 5 CONCLUSION

Overall, this study shows that attractiveness of human face increases when it appears with other faces than appears alone, and the mechanisms are distinctive when compared with higher or lower attractive faces. The emotional brain system plays an important role in the process of facial attractiveness, which can be moderated by personalized narcissism. These findings have important implications for understanding how we judge facial attractiveness and make social interactions. Our work is of great significance for individuals to improve their self‐confidence in the cognition of their facial attractiveness.

## FUNDING

National Natural Science Foundation of China (31920103009, 31871137, and 32071100); the Major Project of National Social Science Foundation (20&ZD153, 19ZDA363); Young Elite Scientists Sponsorship Program by China Association for Science and Technology (YESS20180158); Science and Technology Planning Project of Guangdong Province of China (2019A050510048); Guangdong Key Basic Research (grant 2018B030332001); Natural Science Foundation of Guangdong Province (2020A1515011394); Shenzhen‐Hong Kong Institute of Brain Science‐Shenzhen Fundamental Research Institutions (2019SHIBS0003); and Shenzhen Science and Technology Research Funding Program (JCYJ20180507183500566 and JCYJ20190808121415365).

## CONFLICT OF INTEREST

The authors declare no conflicts of interest.

### PEER REVIEW

The peer review history for this article is available at https://publons.com/publon/10.1002/brb3.2561

## Data Availability

Some or all data or code generated or used during the study are available from the corresponding author by request.
